# Delivery of evidence-based interventions for psychological distress in persons living with IBD: a roadmap for human-centred design and implementation

**DOI:** 10.1093/jcag/gwaf024

**Published:** 2025-09-30

**Authors:** Maria MacDonald, Courtney Heisler, Natalie Willett, Noelle Rohatinsky, Sophie Farina, Michael Stewart, Michael Vallis, Tiffany Shepherd, Barbara Currie, Jessica Robar, Thea Huard, Emily Neil, Jennifer L Jones

**Affiliations:** Dalhousie, Internal Medicine, Nova Scotia Health, Halifax, B3H 4R2, Canada; Digestive Care & Endoscopy, Dalhousie, Nova Scotia Health, Halifax, B3H 4R2, Canada; Digestive Care & Endoscopy, Dalhousie, Nova Scotia Health, Halifax, B3H 4R2, Canada; University of Saskatchewan, Saskatoon, S7N 5A2, Canada; Nova Scotia Health, Halifax, B3H 4R2, Canada; Digestive Care & Endoscopy, Dalhousie, Nova Scotia Health, Halifax, B3H 4R2, Canada; Dalhousie Family Medicine, Halifax, B3J 3T4, Canada; Department of Psychology and Neuroscience, Halifax, B3H 4R2, Canada; Digestive Care & Endoscopy, Dalhousie, Nova Scotia Health, Halifax, B3H 4R2, Canada; Digestive Care & Endoscopy, Dalhousie, Nova Scotia Health, Halifax, B3H 4R2, Canada; Patient Research Partner, Dalhousie, Halifax, B3H 4R2, Canada; Patient Research Partner, Dalhousie, Halifax, B3H 4R2, Canada; Digestive Care & Endoscopy, Dalhousie, Nova Scotia Health, Halifax, B3H 4R2, Canada

**Keywords:** IBD-related psychological distress, mental health, community supports, inflammatory bowel disease (IBD)

## Abstract

**Background:**

Inflammatory bowel disease-related psychological distress (IBD-PD) refers to the emotional impact of IBD and has been shown to be associated with increased disease severity, comorbid mental health disorders, and increased mortality.

**Aims:**

This study aims to identify the facilitators for accessing evidence-based interventions for IBD-PD to inform the design and implementation of patient-centred models for IBD mental health support in the future.

**Methods:**

This was a qualitative research study in which a semistructured interview script was developed by a multidisciplinary team guided by the domains of the COM-B Behaviour Change Wheel framework. Using thematic analysis, codes were generated to identify themes using an inductive approach.

**Results:**

Fourteen participants were successfully recruited (*n* = 14). Thematic analyses identified the following major themes: (1) mental health should be treated as an integrated component of specialty IBD care; (2) use of self-help strategies alongside existing supports is feasible, acceptable, and accessible; (3) accessing support for IBD-PD through virtual care is often acceptable; and (4) flexible, multifaceted delivery models for IBD-PD are needed. All participants felt that mental health should be discussed at IBD clinic visits. Preferences for hybrid formats for IBD-PD care were clear. Most participants felt that it was important for psychological support persons to also have IBD knowledge. Participants felt strongly that a more qualified psychologist, even in the absence of IBD knowledge, was their top priority**.**

**Conclusions:**

Some key facilitators identified from this study include healthcare professionals discussing IBD-PD directly with their patients, offering hybrid formats for IBD-PD, and integrating self-help strategies into care.

## Introduction

Rising inflammatory bowel disease (IBD) prevalence and management complexity continue to expand year-over-year.^1^ This decade, it is projected that an estimated 470 000 Canadians will be diagnosed with IBD.^2^ Canada has the world’s highest observed prevalence rates of IBD, with the eastern provinces reporting the highest rates in the country.^3^

Inflammatory bowel disease-related psychological distress (IBD-PD) refers to the emotional reactions to a chronic disease like IBD, which can be associated with increased comorbid mental health disorders, and symptoms of active disease.^4^^,^^5^ IBD-PD is also associated with increased primary care and Emergency Department (ED) visits.^6^

The COVID-19 pandemic shone a spotlight on the growing mental health crisis in the general population and in those with IBD.^7^ In the IBD population alone, previous estimates have found that the majority of IBD patients experience some form of IBD-PD at some point throughout their IBD journey, and nearly half of IBD patients felt that there was inadequate access to mental health supports for IBD-PD.^8^ These findings are similar to those observed in other published studies.^9^^,^^10^ Despite this lack of access, surveyed IBD patients have indicated high levels of comfort with discussing psychological distress with their IBD care providers or team (including gastroenterologists or IBD nurses) compared to receiving support from formalized mental health professionals.^11–13^

Evidence from systematic reviews and meta-analyses demonstrates that psychological interventions, particularly cognitive-behavioural therapy, psychoeducation, and mindfulness, produce small but clinically meaningful improvements in quality of life, depression, and anxiety in adults with IBD. Multimodal and group-based interventions, as well as integrated care models that embed psychological support within IBD clinics, are associated with higher patient engagement and improved outcomes compared to external referral pathways. Research on rheumatological chronic diseases emphasizes the importance of integrating psychological support into standard clinical care in a manner that aligns with each patient’s unique biopsychosocial needs.^14^^,^^15^

Incorporating psychological support into clinical practice involves routine screening, direct access to in-service psychological care, and multidisciplinary collaboration. In prior studies, patients have expressed a preference for integrated, accessible psychological services and value the validation of their symptoms and emotional well-being as part of the treat-to-target approach.^14^ This study describes the results of patient preference for facilitators of mental health interventions for IBD-PD to improve the delivery of IBD-PD-related care.

### Aim

This research initiative aimed to identify patient-perceived facilitators to accessing evidence-based mental health support for IBD-PD. This information will inform the design and implementation of patient-centred interventions and strategies.

## Methods

### Participants and interviews

**Figure 1.**
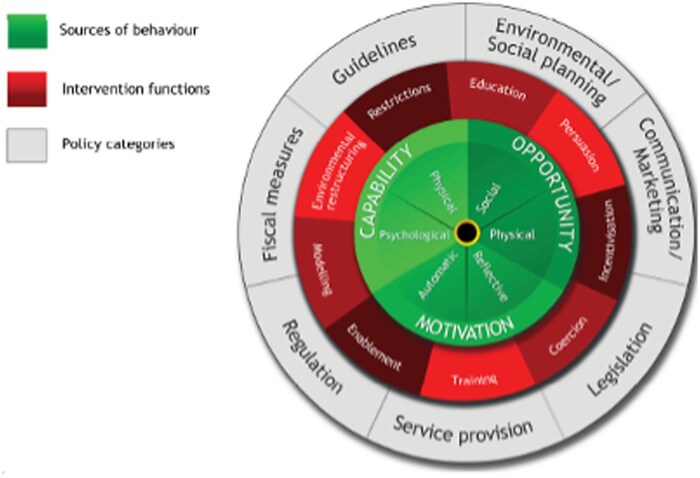
COM-B Behaviour Change Wheel.

Adults diagnosed with IBD were recruited using purposeful sampling from IBD clinics at the QEII Health Sciences Centre in Halifax, Nova Scotia via email or social media campaigns. Interview scripts were developed in collaboration with physicians, nurses, patient research partners, and researchers with a mutual interest in IBD-PD. Question development was informed by the theoretical domains framework (TDF) and the COM-B Behaviour Change Wheel framework, 2 validated, determinate implementation science frameworks.^16^^,^^17^ The TDF is a multidisciplinary framework synthesized from different behaviour change theories used to assess the implementation of a behavioural change intervention and identify barriers and facilitators to behaviours targeted within that intervention.^16^ The COM-B Behaviour Change Wheel framework involves 3 essential conditions (capability, opportunity, and motivation) and sees them as an interacting system where behaviours can be modified through intervention functions (ie, training, incentivization, and policies) that target one or more of the 3 conditions (Figure 1).^17^

During the 1-hour interview, participants were asked about their experiences, the impact, and the causes of psychological distress related to their IBD as well as facilitators and preferences related to access to psychological support services throughout their disease course. This did not include a pilot test; no repeat interviews were carried out and interview scripts were not returned to the participants for feedback. Interviews were cofacilitated by a researcher and patient research partner only. All audio from each interview was recorded on the Zoom for Healthcare platform. Audio was transcribed using software and cleaned by research assistants. All study activities and protocols were approved by the NSH Research Ethics Board prior to study commencement.

### Analysis

The initial themes and coding schemes were developed while reviewing hard copies of the first 4 transcripts. Themes and subthemes were created using an inductive coding approach by the research team. Transcripts were then uploaded into NVivo Pro (Version 12), and each interview was coded by topic to further refine the initial coding following the thematic analysis process outlined by Braun and Clarke.^17^

Psychological support settings coded discussions as they related to group settings, psychological support from health care providers, informal support systems, one-on-one settings (with both IBD practitioners and clinical psychologists), peers, and self-help. Questions about virtual care were first coded separately and then also coded to psychological support contexts when necessary.

## Results

**Figure 2.**
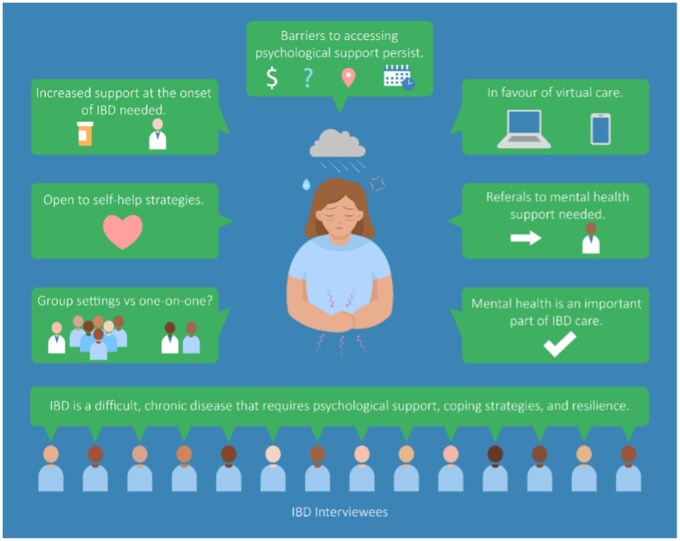
Visual summary of patient-informed themes.

Nineteen individuals were invited to participate in the study, and 14 were successfully interviewed. The mean participant age was 38 years (range 23-57). Most participants were female (57%, or 8/14), and most worked full-time (64%, or 9/14). All had completed at least a high school education. Diagnosis of Crohn’s disease and ulcerative colitis each comprised 29% (4/14) of the sample. The remaining participants had unspecified IBD. No participants identified as a member of a minority group. Twenty-nine percent lived in a rural area (4/14), and 71% (10/14) lived in an urban area. Qualitative thematic analysis identified key themes across participant responses (Figure 2).

### Theme 1: Mental health should be treated as an integrated component of specialty IBD care

Participants strongly supported mental health as an integrated and essential part of IBD care. Participants reflected that the body and mind are connected and not separate entities, and mental stressors can trigger. This reinforces the need for access to IBD-PD supports and services.

Mental health is important for physical health because they’re not separate entities. I think it’s something that really is lacking in all aspects of healthcare. (P11)

### Theme 2. Use of self-help strategies alongside existing supports is feasible, acceptable, and accessible

Participants had a diverse set of supports in place through a combination of informal support networks, one-on-one psychological support, and support groups. Significantly, half of the participants (7/14) said that they did not access formal psychological support services. Instead, this group preferred informal support networks or self-help strategies they already had in place. None of the 7 participants who did not access formal psychological support reported declining an offer; rather, they were either unaware of available services or faced barriers to access. The other half had exposure to formal support. Three participants indicated that they would not be willing to bring up mental health with their physician during a visit because they were concerned it would compromise their care or felt intimidated to bring it up due to the stigma that they felt was attached to IBD-PD. Otherwise, all other participants felt comfortable raising their mental health concerns with a physician.

Of those participants who were asked about how they were made aware of resources (either as a referral or whether they found resources on their own), 5 participants said that they were aware of resources or referred to resources by their healthcare providers, including social workers, counselling services (through a partner’s work, University, or referral to NSH mental health and addiction services). Six participants said that they were not aware of psychological support and that they did not know where to look.

#### Self-help, information, and existing resources

Overall, participants who responded directly with their preference for a web or mobile-based application versus a workbook or paper resources preferred using an application (App^7^ Workbook^3^) Seven participants discussed being proficient and able to find the resources and information they needed to help themselves cope and learn about IBD.

#### Informal supports

Although half of the participants accessed only informal support, 10 of 14 discussed how informal support through family, friends, and peers was essential to their overall care. Some participants accessed these informal networks less and felt that worrying others about their symptoms would be burdensome.

My whole family, especially my mother, has been one of my biggest supports. They make up the biggest part of my support system. (P07)

#### Importance of IBD knowledge in addition to psychological supports

Twelve participants felt that it was important for psychological support to also have IBD knowledge. However, these preferences were sometimes expressed alongside concerns about access, and some participants felt strongly that a more qualified psychologist, even in the absence of IBD knowledge, was their top priority**.**

Something is better than nothing. If an individual needs support, which comes in the form of someone who doesn’t have GI knowledge, it’s still better than nothing. (P10)

### Theme 3. Accessing support for IBD-PD through virtual care is often acceptable

Two participants indicated that they would prefer in-person care to virtual care, and 1 was undecided. Most participants (11/14) were comfortable with virtual care. Of those who preferred virtual care, 2 indicated that some form of hybrid setup would be preferable to exclusively virtual care.

#### Benefits of virtual care

Participants felt that virtual care helped them avoid negative associations with healthcare environments, provided ease of access and privacy, and provided better choice and availability of providers. When prompted to discuss concerns and benefits of virtual care, some participants indicated that they felt that virtual care allowed them to access care from their homes privately.

Virtual care is convenient. You don’t have to worry about travel. Especially specific to IBD, there are a lot of times you really don’t feel like leaving your house, or you feel like you can’t leave your house. (P01)

#### Concerns with virtual care

Only 2 participants brought up concerns about virtual care. The first concern is the inherent risks of moving to a virtual environment where data security presents risks for confidentiality. While many participants felt that virtual environments offered more privacy and were freeing, these 2 participants felt that something is lost in the virtual environment and that there is no replacement for the immediate feedback and body language that can be part of in-person psychological support.

One disadvantage with the internet is there could be a risk of a breach of confidentiality over the internet compared to in person. (P07)

#### Motivation to use virtual care

Participants expressed that they would be motivated to try virtual care if it were urgent, reduced travel times, made it easier to book flexibly, or was the only option, like it was during the COVID-19 pandemic. Two participants pointed out that the steps and information required to access virtual support need to be provided by someone in their healthcare team. One participant suggested that they would be more motivated to access virtual care in a group setting.

### Theme 4: Flexible, multifaceted delivery models for IBD-PD are needed

Preferences for a range of psychological support formats were clear, and no single approach or setting was acceptable for everyone.

#### Group settings

Some participants felt a group session would be beneficial, while others felt that hearing about more severe forms of IBD would be distressing in a group setting. When asked about having a session with a psychologist or both an IBD practitioner and a psychologist in the group setting, 4 patients agreed that having both would be their preference. Five participants were somewhat ambivalent about a group setting without a clear preference.

While it would be nice to talk to people in a group in a similar situation, I would be concerned they would feel anxious and worried when hearing other people’s symptoms as it might relate to their potential symptoms. (P11)

#### Preference for group settings

The participants who had a clear preference for group settings felt that it fostered an environment of empathy while helping to normalize the experience of IBD and decrease social isolation and stigmatization.

I'm comfortable with group sessions because you can use advice from shared experiences and help you feel less alone. (P12)

#### Concerns with group settings

One participant felt that they would be better served discussing specific coping strategies with someone one-on-one. A few participants expressed concerns that those at the beginning of their journey or with less severe symptoms may be hesitant to go to a group session where they will hear about more difficult manifestations of the disease from others.

I don’t know that I want to share some of the difficulties that I face with strangers who are just trying to do their own coping and have no answers for me. (P03)

#### One-on-one sessions

Some participants clearly preferred one-on-one sessions, feeling that they would like to talk to the most qualified person. One participant felt that the comfort level with the provider would play a role in continuing to receive care in a one-on-one setting.

#### Preference for one-on-one sessions

One-on-one sessions with a counsellor or psychologist were thought to be more effective than group sessions because they would allow patients to talk to the most qualified professional freely and receive specialized care while ensuring confidentiality. Overall, there was strong support for one-on-one sessions in the interviews. Many participants were willing to use a one-on-one session but lacked the information, access, or means to do so.

I would prefer somebody who is dedicated to the brain for the same reason that I would prefer a GI over a GP. So, a psychologist or a psychiatrist would be preferential to me over a general physician. (P09)

#### Concerns with one-on-one sessions

Participants did not raise serious concerns about one-on-one sessions. Still, when asked about the effectiveness of one-on-one psychological support, they responded that it was a matter of preference, access, and a trade-off with the benefits of group settings. There was overlap among participants who supported group and one-on-one settings, with some indicating that the best outcome would be having a combination of supports instead of using only one setting exclusively.

#### Peer support

Eleven participants expressed apparent openness and support for peer support; however, support came with a strong caveat from most who supported peer support: The facilitator should have adequate training. Provided that the peer support mentor was trained to deliver the support and facilitate interactions, participants were open to trying a peer support program.

## Discussion

This study focused on identifying facilitators for accessing IBD-PD care. It is helpful to map these results to the 3 components of the COM-B framework (Table 1).^15^ In this study, the target behaviour of focus is the IBD patient accessing evidence-based care for IBD-PD. Capability refers to the individual’s psychological and physical capacity to engage in the target behaviour.^16^ Opportunity refers to the factors outside the individual that make the behaviour possible.^16^ Motivation refers to the psychological processes (conscious and unconscious) that energize and direct behaviour.^16^ Possible facilitators in accessing care for their IBD-PD span across all 3 conditions of the COM-B framework, and each can encourage or enable the target behaviour to occur in these different ways.

**Table 1.** Themes and subthemes related to facilitators for the COM-B domains.

Theme	Subtheme (s)	COM-B domain	Intervention functions
1) Mental health should be treated as an integrated component of specialty IBD care		Capability Opportunity	Enablement Training
2) Use of self-help strategies alongside existing supports is feasible, acceptable, and accessible	Self-help, information, and existing resources Informal supports Importance of IBD knowledge in addition to psychological supports	Capability Opportunity Motivation	
3) Accessing support for IBD-PD through virtual care is often acceptable	Benefits of virtual care Concerns with virtual care Motivation to use virtual care	Capability Opportunity Motivation	Training Enablement Environmental restructuring
4) Flexible, multifaceted delivery models for IBD-PD are needed	Group settings Preference for group settings Concern with group settings One-on-one sessions Preference for one-on-one sessions Concerns with one-on-one settings Peer support	Capability Motivation	Education Enablement Training

The themes and subthemes related to the opportunity component of COM-B include Theme 1: Mental health should be treated as an integrated component of specialty IBD care. In our study, participants felt that mental health should be discussed at IBD clinic visits. A study completed in 2017 found that most healthcare providers did not discuss how IBD may affect the quality of life and overall mental health of patients during routine visits.^18^ This is reflected in our study, as only 5 out of 14 participants were referred to mental health support by their healthcare provider. This highlights that healthcare providers should initiate the conversation around mental health to ensure patients feel safe sharing their experiences in a supportive environment.

The themes and subthemes related to the capability component of COM-B include Theme 4: Flexible, multifaceted delivery models for IBD-PD are needed. Overall, our study found that no single approach fits all participants. While some participants appreciated the validation and normalization that group settings could offer, others found the idea distressing or unhelpful, particularly if hearing about more severe disease experiences. This divergence in preference suggests that group settings may not be universally acceptable and may require careful triaging or opt-in structures to ensure that participants are matched to their preferred support format. Similar preference differences were observed with virtual versus one-to-one care. These examples highlight the importance of listening to the patients and addressing their unique needs when delivering care for IBD-PD.

Understanding what motivates an individual to participate in IBD-PD support should determine what services to offer as seeking the right resources for each patient can positively impact patients living with IBD.^19^^,^^20^ The themes and subthemes for facilitators related to the motivation component of COM-B include Theme 2: Use of self-help strategies alongside existing supports is feasible, acceptable, and accessible. In this study, participants clearly preferred an online application for self-help strategies. They also discussed how informal support through family, friends, and peers was important to their overall care. IBD impacts the patients and family members in terms of their interpersonal relationships.^21^ As healthcare providers, it is essential to recognize this and be ready to support family dynamics when appropriate.

### Intervention functions and implementation strategy

The mode of delivery is an essential aspect of intervention design. The use of virtual platforms has rapidly increased as an effective tool that allows for privacy and enables avoidance of travel and triggering hospital environments. Two participants were concerned with the loss of human connection with virtual care. Healthcare professionals who deliver virtual IBD-PD support must recognize these challenges and implement changes to make virtual care more comforting for patients.^21^ For example, limiting background noises and distractions during virtual care delivery can improve the virtual experience for patients.^20^ These findings suggest that virtual and in-person care may be utilized as a facilitator in IBD-PD care and provide more evidence for implementing a patient-focused preference for IBD-PD support.

In practical terms, for the implementation of the findings in this study, IBD clinics could adopt a stepwise “patient-first” framework, beginning with universal mental health screening at routine visits, followed by offering patients a menu of options, including self-help resources, virtual one-on-one therapy, in-person therapy, or peer-facilitated groups. Patients could be triaged based on a brief preference assessment, existing self-management behaviours, and access constraints (eg, tech access or travel capabilities).

In Canada’s provincial healthcare context, resource allocation varies widely. Given this heterogeneity, a “modular approach,” starting with self-directed tools (print, app), stepping up to virtual or in-person formats based on patient choice and need, and integrating peer-mentorship guided by brief training programs, offers the scalability needed across large (eg, Nova Scotia Health) and small regional centres. This modular approach allows for resource allocation tailored to local capacity while still honouring patient-centred principles. Implementation of such models would benefit from health system investment in training, digital infrastructure, and integration of mental health professionals into IBD care teams.

### Strengths and limitations

The strengths of this study include participants from different age groups, genders, geographic locations, and disease types. Although 6 participants had unspecified IBD, this reflects limitations in clinical documentation where subtype (Crohn’s vs ulercerative colitis) was not clearly recorded. Given our focus on psychological support rather than disease-specific outcomes, their inclusion was considered appropriate. The interview questions involved iterative feedback from researchers, IBD care providers, and patient research partners.

This study has key limitations. The sample of interviewees lacked ethnic diversity, and researchers faced difficulty in recruitment from key marginalized groups. The sample was local to Nova Scotia, so the generalizability of these findings does not necessarily extend to the rest of Canada or other populations.

## Conclusion

This study highlighted the need for integrating a combination of mental health services within IBD care and the need for increased awareness of the impact that IBD can have on a patient’s quality of life. It is essential to ensure that the design of future interventions aligns with patient-specific needs and that patients receive appropriate, high-quality, personalized care for their IBD-PD.

## Supplementary Material

gwaf024_Supplementary_Data

## Data Availability

The data that support the findings of this study are not openly available due to reasons of maintaining confidentially for participants.
